# A Novel Distinct Genetic Variant of Tomato Torrado Virus with Substantially Shorter RNA1-Specific 3’Untranslated Region (3’UTR)

**DOI:** 10.3390/plants10112454

**Published:** 2021-11-13

**Authors:** Marta Budziszewska, Przemysław Wieczorek

**Affiliations:** Department of Molecular Biology and Biotechnology, Institute of Plant Protection—National Research Institute, Wegorka 20 Street, 60-318 Poznan, Poland

**Keywords:** tomato torrado virus, genetic variability, 3’untranslated regions, tandem repeats

## Abstract

Tomato torrado virus (ToTV) induces severe systemic necrosis in *Solanum lycopersicum*. This work aimed at describing the genetic variability of necrosis-inducing ToTV-Wal’17 collected in 2017, derived from the ToTV-Wal’03 after long-term passages in plants. Sequence analyses of the ToTV-Wal’17 indicated twenty-eight single nucleotide substitutions in coding sequence of both RNAs, twelve of which resulted in amino acid changes in viral polyproteins. Moreover the sequencing data revealed that the 3’UTR of ToTV-Wal’17 RNA1 was 394 nts shorter in comparison to Wal’03. The performed sequence analyses revealed that 3’UTR of RNA1 of ToTV-Wal’17 is the most divergent across all previously described European isolates.

## 1. Introduction

Tomato torrado virus (ToTV) is a member of the *Torradovirus* genus in the *Secoviridae* family [1,2]. In its natural environment, ToTV infects *Solanum lycopersicum* causing “torrado” disease, manifested by burn-like severe necrosis of stems, leaves, and fruits [3,4,5]. In the field or greenhouses, the virus is transmitted by whiteflies [3,6]. Tomato torrado virus has been identified in Spain [7], Hungary [8], Poland [6], Canary Islands [9], France [10], Panama [11], Italy [12], Australia [13], Colombia [14], and Morocco [15]. From 2009–2013, the ToTV was included on the EPPO Alert List (https://gd.eppo.int/taxon/TOTV00/documents accessed on 12 February 2009) due to its expansion and severity. Recently, the ToTV was found to be infecting tomato crops in new locations, South Africa [16] and Serbia [17], indicating that the virus should still be considered as a serious threat to tomato. Under experimental conditions, ToTV can be mechanically inoculated to, among others, *S. lycopersicum* or *N. benthamiana* [4,18], a well-known model for plant–pathogen interactions [19].

The ToTV has a bipartite genome with RNA1 (7802–7814 nucleotides, excluding polyA tail) and RNA2 (5390 nucleotides, excluding polyA tail). Coding sequences in RNA1 and RNA2 are flanked with untranslated regions (UTRs). The 5’UTRs of RNA1 and RNA2 of ToTV are composed of 107 nts and 181 nts, respectively, and remain genetically conserved between described ToTV isolates. Compared to the known RNA plant viruses, ToTV, like other torradoviruses, has very long 3’UTRs in both genomic strands [3,4]. The 3’UTR in RNA1 (1230 nts) has a variable region (VR) of 241 nts followed by 989 nts of the conserved region (CR); the 3’UTR in ToTV RNA2 is shorter (1106 nts) and begins with 103 nts followed by CR sequence having 98% similarity with the corresponding region from RNA1. Noteworthy, within the CR in both RNA1 and RNA2, two direct repeats were identified, D1a/D1b and D2a/D2b, arranged in the following order: D1b-D2a-D1a-D2b [20]. Notably, described full-length sequences of the Polish ToTV Kra and Ros isolates revealed remarkable length heterogeneity in VR that arose from nucleotide deletions in this region [20,21].

RNA viruses are known to have a high mutation rate because virus RNA polymerase lacks proofreading activity. Thus, during each replication cycle, a pool of mutated RNA species is generated, and in particular environmental conditions, only the optimal RNA master sequence will preferentially accumulate. This is one of the well-known mechanisms of the emergence of virus genetic variability [22].

In this work, we assessed the genetic variability of new ToTV isolate, the ToTV-Wal’17 originated from the previously described ToTV-Wal’03, causing severe necrosis on tomato and leaf malformations on *N. benthamiana*. The ToTV-Wal’03 was mechanically passaged spontaneously over 13 years and isolated from *S. lycopersicum* in 2017 for comparative genetic analyses. Here, we showed that apart from the several new point mutations identified in the ToTV-Wal’17 genome, the ToTV-Wal’17 has significantly shorter RNA1 3’UTR and, as such, still remained highly infectious to tomato. Compared to previously described ToTV isolates, the mentioned RNA1 3’UTR of ToTV-Wal’17 was described to be shorter of 394 nts in the CR region. This, in turn, significantly changed the conserved stretch of the D1b-D2a-D1a-D2b sequences. The performed analyses pointed that in the context of 3’UTR in RNA1, the ToTV-Wal’17 is the most diverse across all previously described European isolates of ToTV.

## 2. Materials and Methods

The ToTV-Wal′03 [4] was used as a virus source. The virus was maintained and propagated on *N. benthamiana* or alternately in *S. lycopersicum* plants, periodically collected, stored at −20 °C, and used as the source of virus inocula for 13 years [5]. In 2017, RNA was isolated from the infected plants (*S. lycopersicum*) virus and named hereafter ToTV-Wal’17.

The total RNA was extracted from diseased tomato plants using TriReagent (Thermo Fisher Scientific, Lenexa, KS, USA) [19] and cDNA was synthesised in the presence of the asTo2C_pJL_RV primer [18] and SuperScript IV Reverse Transcriptase (Thermo Fisher Scientific, Lenexa, KS, USA) following the manufacturer’s instructions. The cDNA, covering the full-length RNA1 and RNA2, was amplified utilising polymerase chain reaction with CloneAmp HiFi PCR Premix (Takara, Kusatsu, Shiga, Japan) and primers asTo1A_pJL_FW/asTo2C_pJL_RV (RNA1) and asTo2A_pJL_FW/asTo2C_pJL_RV (RNA2) [18]. The cDNA copies were fused with pJL89 plasmid (NEBuilder HiFi DNA Assembly Master Mix; New England Biolabs, Ipswich, MA, USA) and cloned in *E. coli* Stellar competent cells (Takara, Kusatsu, Shiga, Japan). Then, the recombined plasmids were Sanger sequenced to generate overlapping contigs. The retrieved contig sequences were assembled using BioEdit software [23], and the obtained consensus sequences of RNA1 and RNA2 were analysed.

The sequences of Wal’17 isolate were aligned with genomic sequences of Wal′03 (EU563948, EU563947) using Basic Local Alignment Search Tool (https://blast.ncbi.nlm.nih.gov/Blast.cgi accessed on 11 October 2008). Additionally, the multiple nucleotides and amino acid sequence analyses of all known ToTVs isolates were aligned using MEGAX software [24], followed by the phylogenetic studies [13], a maximum-likelihood algorithm, and 1000 bootstrap value.

The recombinant plasmids pJL89-Wal’17-RNA1 and pJL89-Wal’17-RNA2 were each introduced by transformation into *A. tumefaciens* GV3101. Subsequently, single positive bacteria colonies were picked and grown individually in liquid LB medium (supplemented with 50 mg/L rifampicin and 100 mg/L kanamycin) for 48 h at 28 °C. The agroinfiltration of 4-week-old seedlings of *S. lycopersicum* was carried out as previously described [19]. Additionally, the tomato plants were agroinfiltrated with infection clones of ToTV-Kra previously described by Wieczorek et al. (the ToTV-Kra was found to be the most similar phylogenetically to the ToTV-Wal’03) [19]. The agroinfiltrated plants were maintained in a greenhouse with photoperiod and temperature of 16 h-28 °C/8 h-24 °C (day/night). Visual symptoms were monitored ten days after infiltration and the plant material (two discs of two leaves, ~5 mm in diameter) was collected for subsequent qualitative and quantitative analyses. For this, the total RNA was isolated using TriReagent (Thermo Fisher Scientific, Lenexa, KS, USA) followed by DNase treatment and then the equal amount of the RNA was converted into cDNA using 200U RevertAid Transcriptase (Thermo Fisher Scientific, Lenexa, KS, USA) with random primers. Subsequently, RT-PCR detection of ToTV was carried out using 1× DreamTaq PCR Master Mix (Thermo Fisher Scintific, Lenexa, KS, USA), 500 nM forward and reverse primers (5′AAGGAAAATGACCATGGGGTCCA3′/5′CCCACTAGTTTTTTTTTTTTTTTTTTTTAAAATACAT3′) and 1 µL of cDNA. Additionally, the virus accumulation was performed in a RT-qPCR using 1× iTaq SYBR Green master mix (Bio-Rad, Hercules, CA, USA), 500 nM mixture of primers complementary to RdRp in ToTV RNA1 (5′GGAAAAGGTTTTTGGGAAGC 3′/5′CATCAGACTGGCGGAAAAAT3′) or Vp35 encoded by ToTV RNA2 (5′TGCTGAGGTGCTATCACTGG3′/5′ACCCATGCCGGAAATATACA3′) and 1 µL of the prepared cDNA. For absolute quantification of ToTV RNA1 and RNA2, the obtained Ct values were plotted against the external standard curves prepared using 5-fold serial dilutions of plasmids containing cDNA copies of RNA1 or RNA2. All the reactions were carried out in triplicates.

## 3. Results and Discussion

The obtained complete sequences of RNA1 and RNA2 of ToTV-Wal’17 have been deposited in the NCBI database (MW729382 and MW729383). The lengths of RNA1 and RNA2 of the ToTV-Wal’17 were calculated and showed to have 7419 and 5390 nts (excluding polyA tail), respectively, indicating that RNA1 of Wal’17 was 394 nts shorter than the corresponding RNA strand from ToTV-Wal’03. The performed comparative sequence analyses of RNA1 of ToTV-Wal’17 showed 99% (in a range of 1–6826 nts), and 77% (6899–7419 nts) sequence identity within the corresponding RNA of ToTV-Wal’03 (EU563948), respectively. On the other hand, the entire RNA2 had 99% sequence identity with the corresponding RNA of Wal’03 (EU563947). All indicated nucleotide substitutions in both RNAs of Wal’17 were listed in Table 1.

### 3.1. Analysis of 5’Untranslated Regions (5’UTRs)

Analysis of ToTV-Wal’17 RNA1 and RNA2 5’UTRs showed that the regions had the same length and nucleotide sequences as the 5’UTRs from the ToTV-Wal’03 [4].

### 3.2. Analysis of Coding Sequences (CDS)

The coding sequences of ToTV-Wal’17 had the same length as in ToTV-Wal’03 and comprised 6476 and 4116 nts in RNA1 and RNA2, respectively. In RNA1, 22 nucleotide substitutions were identified, of which eight of them changed the amino acid. Concerning RNA2, six nucleotide substitutions were indicated in CDS, and four of them resulted in amino acids substitution. All the described mutations in CDS were listed in Table 1.

### 3.3. Analysis of 3’Untranslated Regions (3’UTRs)

The RNA1 and RNA2 3’UTRs of ToTV-Wal’17 had 836 and 1092 nts, respectively. In general, in all ToTV isolates sequenced so far, the 3’UTR in RNA1 was longer than in RNA2. Surprisingly, contrary to ToTV-Wal’03, the 3’UTR in RNA1 of ToTV-Wal’17 was 256 nts shorter than that in RNA2. Moreover, by comparing sequence length, it was shown that 3’UTR in RNA1 of ToTV-Wal’17 was 394 nts shorter (32%) than the corresponding sequence from ToTV-Wal’03. On the other hand, the RNA2 3’UTR of ToTV-Wal’17 was the same in length as the analogous sequence from ToTV-Wal’03.

Previously, it was shown that 3’UTR in ToTV RNAs were predicted to form structured regions [20]. Additionally, RNA1 and RNA2 3’UTRs of European isolates of ToTV are generally long (over 1000 nucleotides), and they are organised according to the following scheme: stop codon, followed by variable region (D-VR, 241 and 103 nts in RNA1 and RNA2, respectively), then conserved region (D-CR, 989 nts in RNA1 and RNA2) with terminal polyA tail. Moreover, in the CR in the 3’UTRs, direct repeats were identified and arranged in the following repetitive motifs: D1b–D2a–D1a–D2b [20].

**Figure 1:**
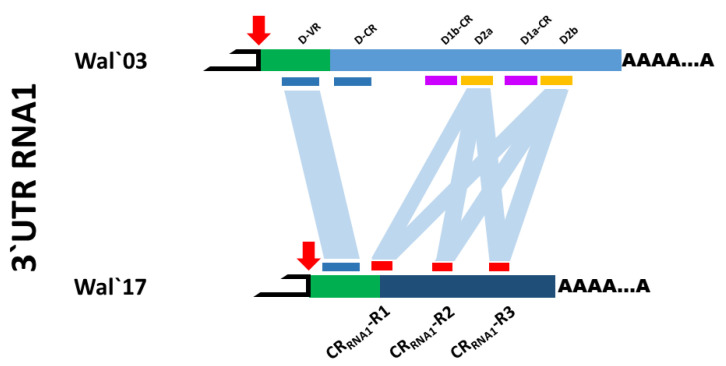
Schematic comparison of 3’untranslated regions of RNA1 of ToTV-Wal’03, and ToTV-Wal’17. The repetitive motifs were indicated with blue (D-VR, D-CR), violet (D1b-CR, D1a-CR), orange (D2a, D2b) (ToTV-Wal’03), and red (ToTV-Wal’17) bars. The red arrow marks the stop codon. The green blocks are common 3’UTR regions for both isolates. The D-VR D-CR regions (the blue bars) are present together only in the genome of ToTV-Wal’03. In RNA1 3’UTR of ToTV-Wal’17 only the D-VR region was identified.

Concerning the ToTV-Wal’17, sequence comparison of its RNA1 3’UTR indicated a short variable region D-VR was preserved in the viral RNA. However, the D-CR region in RNA1 shared only 77% identity with the corresponding D-CR region from ToTV-Wal’03. Due to the high sequence variability, the repeated sequences, D1b–D2a–D1a–D2b were not identified. Notably, within the D-CR of the 3’UTR of RNA1 in ToTV-Wal’17, three other repetitive sequences of 70–71 nts in length (designed as CR_RNA1_-R1 (position 6807–6877), CR_RNA1_-R2 (6899–6969), CR_RNA1_-R3 (7171–7240)) were identified (Figure 1). The three mentioned repetitive sequences share 90–96% identity with the D2b region ToTV-Wal’03 [20,21].

The RNA2 3’UTR of ToTV-Wal’17 shares 99% identity with the corresponding region of ToTV-Wal’03 and in the CR region, the repetitive motifs D1b–D2a–D1a–D2b were still present.

**Figure 2:**
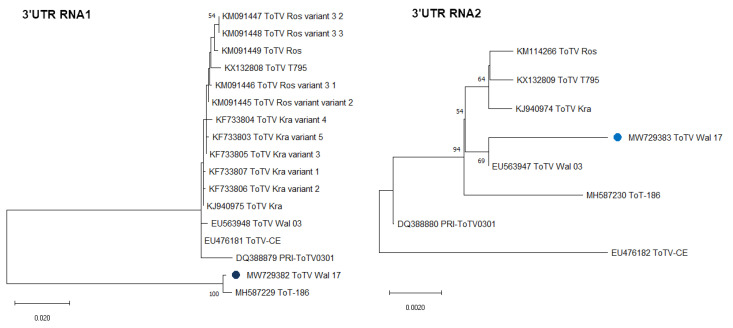
The phylogenetic analyses of 3’untranslated region of RNA1 and RNA2 of ToTV Wal’17. The dendrograms were generated and visualised in MEGA X software, using the maximum likelihood method and 1000 bootstrap value. The scale bars represent a genetic distance. A blue dot indicates the Wal’17 isolate. The accession numbers of sequences deposited in the GenBank NCBI used in analyses are indicated in the trees.

Additionally, performed phylogenetic studies based on the 3’UTR of RNA1 and RNA2 confirmed the results from comparative sequences studies mentioned above. In the dendrogram generated based on 3’UTR RNA1, we showed that the ToTV-Wal’17 isolate clustered with the recently reported South African ToTV isolate, and they both are phylogenetically distant from the other Polish and Spanish isolates of ToTV. The 3’UTR of RNA2 sequence analysis showed that the ToTV-Wal’17 clustered together with the ToTV-Wal’03 and had a short evolutionary distance with other Polish and Spanish isolates (Figure 2).

Here we characterised new mutations (silent and missense) accumulated in the ToTV-Wal’17 genome after mechanical passages from host-to-host over time. We observed that the performed mechanical transmission of the virus did not affect its pathogenicity: neither attenuated nor boosted virus severity on *S. lycopersicum* or *N. benthamiana*.

The high mutation rate of plant RNA viruses is directed by virus RNA polymerase without proofreading activity [22]. However, environmental pressure favours such viral RNA molecules (genomes) that can efficiently replicate, infect and spread in the host [22]. Therefore, it is not surprising that a few new mutations changing amino acid context in virus polyproteins were described in the coding region of the ToTV-Wal’17 genome after 13 years of mechanical passages through the same hosts (tomato and *N. benthamiana*). However, what might be surprising is a new genetic variant of virus RNA1 with substantially shortened 3’UTR. Thus, a significantly shortened sequence and its high variability have not been described so far for other European isolates of ToTV. The comparative sequence studies of 3’UTRs of RNA1 of ToTV isolates deposited in GenBank between 2007 and 2014 showed similar lengths; only in Polish isolates Kra and Ros ToTV were the additional deletion variants observed.

On the other hand, the new ToTV isolate reported in 2020 by Moodley in comparison to the present ToTV-Wal’17 indicates a further reduction in this region of the viral genome. However, in the Wal’17 isolate, the deletions occurred in the D-CR region, which is almost identical in both RNA strands, suggesting that this region might be, to some extent, redundant. Thus, a question arises regarding the reason of significant RNA1 3’UTR shortening in these two geographically distant isolates, including the fact that Wal’17 evolved from a much longer prototype variant of Wal’03.

**Figure 3:**
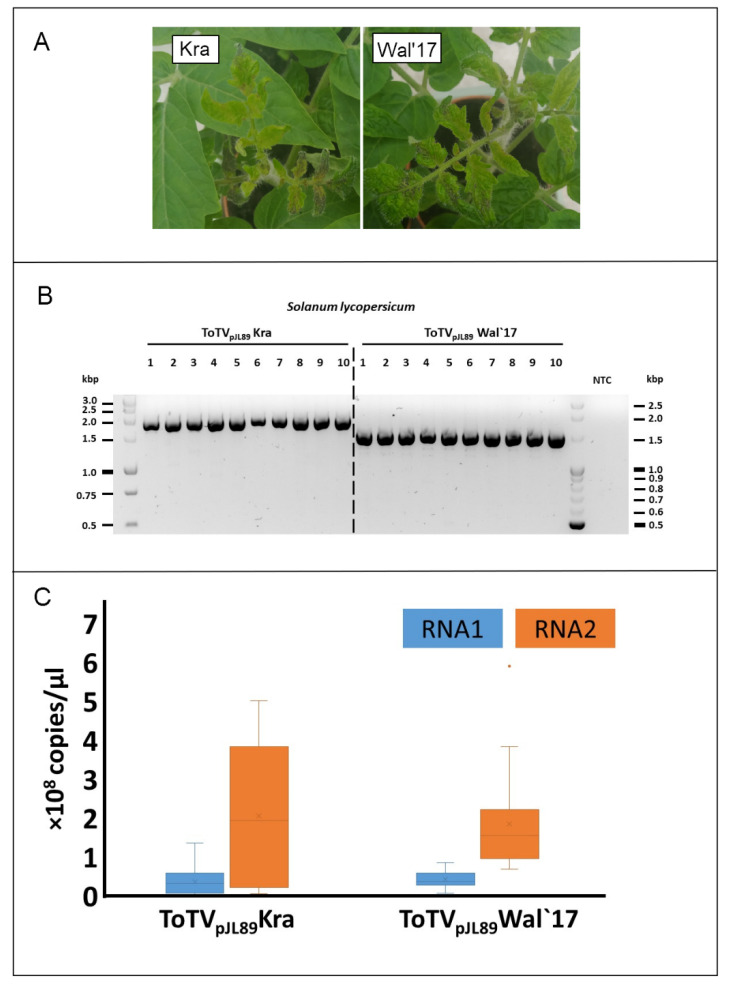
Symptoms induced and virus accumulation of a new isolate of tomato torrado virus isolate Wal’17. Panel (**A**): Necrotic symptoms on *Solanum lycopersicum* plants caused by tomato torrado virus Wal’17 (right) and Kra (left); Panel (**B**): RT-PCR detection differentiating the ToTV-Kra from ToTV-Wal’17 in infected *Solanum lycopersicum*. The different lengths of amplicons arose from variability within the RNA1 3’UTR region in analysed isolates; Panel (**C**): Comparison of the ToTV- Wal’17 and ToTV Kra RNA copies estimated by reverse transcription-quantitative real-time polymerase chain reaction (RT-qPCR).

The region localized at the 3’-termini of positive single stranded viral genomic RNAs is known to contain significant regulatory signals that influence the viral replication process, gene expression, translation and RNA stability. The presence of conserved sequences and their higher order structures, such as cis-acting elements, tRNA-like structures (TLS), stem–loop structures, the poly(A) tail are necessary for the virus life cycle [20]. Interestingly, Yoshida et al. [25] identified unique internal poly(a)tail within 3′UTR region in genomic RNA of tobamoviruses, whereas Guo and Wang in 2019 [26] identified that the length of this region has ranged 77 to 96 nts and may have changed during virus mechanical passaging. Shortening of this region resulted in the reduction of virus accumulation. Rodriguez-Cerezo et al. experimentally proved that 58 nts insertion within 3′ untranslated region of genomic strand, containing four direct repeats, within full length cDNA transcripts of tobacco vein mottling virus was responsible for symptoms attenuation [27]. Therefore, we performed biological test on *S. lycopersicum* infiltrated with *Agrobacterium tumefaciens* transformed with herein obtained recombined plasmids with cloned copies of RNA1 and RNA2 of ToTV-Wal’17. Disease symptoms in plants infected with the ToTV-Wal’17 were compared with symptoms induced on tomato plants infiltrated with *A. tumefaciens* transformed with previously described infectious copies of pJL89-Kra [19] (with an original 3′UTR sequence). The visual symptoms inspection revealed necrotic lesions in all tested plants. Subsequently, the comparative analysis of virus accumulation in tomato infected by ToTV-Wal’17 or ToTV-Kra showed no significant differences in RNA1 and RNA2 level between the aforementioned viruses (Figure 3). This data suggest that the described shortening of the 3′UTR sequence of RNA1 does not involve significant changes in the development of disease symptoms, nor does it significantly affect the level of viral RNA accumulation in the infected plant.

Our previous studies described the genetic variability of 3’UTR in RNA1 of ToTV-Kra and ToTV-Ros, associated with diverse deletions concerning only the D-VR region [20,21]. However, in the mentioned ToTV isolates, the D-CR regions remained relatively unchanged, with preserved repetitive motifs possibly involved in virus adaptation to hosts. It was then discussed that this sequence conservation in D-CR of RNA1 and RNA2 3’UTR fixated the presence of predicted secondary (and possibly tertiary) intramolecular interactions [20] that were anticipated to be created within the RNAs. Structural elements were described in the genome of turnip crinkle virus (TCV) to be responsible for nonsense-mediated decay (NMD)-resistance stabilizing TCV genomic and subgenomic RNAs during replication and expression [28]. Possibly, the short repetitive sequences localized within ToTV 3’UTRs might form similar structured elements involved in stabilizing virus RNAs from NMD as well; however, this aspect will need to be experimentally verified. Budziszewska et al. discussed the phenomenon of genetic heterogeneity in RNA1 of Polish ToTV isolates, suggesting that the variable in length variants of 3’UTRs may have a potential role in the virus replication process and/or transmission by insect vector [20]. One of our hypotheses is that the presence of direct repeats within 3′UTR region of RNA1 in Polish isolates of ToTV may be an effect of virus adaptation to the environment, depending on the presence or lack of the vector—greenhouse whitefly.

The sequencing data and greenhouse observation obtained in the present study suggest that the regulatory elements (involved in virus replication) might be located outwardly in the D-CR region. The mechanism leading to the shortening of RNA1 3’UTR remains unknown; it might have been spontaneous or could have been forced by virus adaptability to the plant host due to long-term passaging (and the lack of insect vector). Further experiments explaining the effect of identified variability in RNA1 and the function of characteristic direct repeats in ToTV 3’UTRs RNAs are necessary to be performed to understand the mechanism of ToTV evolution concerning plant–virus–vector interaction.

## Figures and Tables

**Table 1:** The nucleotide (nt) and amino acid (aa) substitutions in particular functional fragments of RNA1 and RNA2 of tomato torrado virus Wal’17 isolate (MW729382, MW729383) in comparison to the original ToTV-Wal’03 (EU563948, EU563947) sequences.

RNA Strand	Silent Mutations		Missense Mutations	
	Nucleotide change	Functional domain	Nucleotide change (amino acid change)	Functional domain
RNA1	830G > A	Prot	346T > A (V80D)	11K
	1574C > T	Hel		
			1905G > A (D600N); 2058C > T (Y651H); 2242A > G (N712S); 2504G > C (K799N)	Hel
	1964C > T 2216G > A; 2612T > C	Hel/Prot		
	3500A > G; 3734C > T;	Prot/RdRP	3436T > C (V1110A)	Prot/RdRP
	4124T > C 4256T > C 4496C > T	RdRP		
	5603G > A 5810C > T 6101C > T 6473C > T	RdRP/3’UTR		
			4762G > A (R1552K)	RdRP
			5843G > C (Q1912H)	RdRP/3’UTR
RNA2	632G > A	ORF1	1863A > C (N388H); 1865T > C (N388H); 1866C > A (H389N); 2001C > T (L434F)	3A
	2573T > C	Vp35		

Prot—protease; Hel—helicase, RdRp—RNA dependent polymerase, UTR—untranslated region, ORF—open reading frame, 3A—protein involved in virus movement, 11K—11K domain, Vp35 — viral coat protein, subunit 35.

## Data Availability

All data are provided in the manuscript and the sequencing data are deposited in The National Center for Biotechnology Information (NCBI) database.
